# Severe mitral valve regurgitation in a preschool boy following blunt abdominal trauma: a case report

**DOI:** 10.1093/ehjcr/ytaf474

**Published:** 2025-09-19

**Authors:** Samuel Waser, Nagat Elbejou, Matthias Gittermann, Martin Schweiger, Robert Cesnjevar

**Affiliations:** Pediatric Emergency Medicine, Department of Pediatrics, Children’s Hospital Aarau, Tellstrasse 25, 5001 Aarau, Switzerland; Pediatric Emergency Medicine, Department of Pediatrics, Children’s Hospital Aarau, Tellstrasse 25, 5001 Aarau, Switzerland; Pediatric Cardiology, Department of Pediatrics, Children’s Hospital Aarau, Tellstrasse 25, 5001 Aarau, Switzerland; Department of Cardiac Surgery, University Children's Hospital Zurich, Lenggstrasse 30, 8008 Zurich, Switzerland; Department of Cardiac Surgery, University Children's Hospital Zurich, Lenggstrasse 30, 8008 Zurich, Switzerland

**Keywords:** Case report, Mitral valve regurgitation, Flail leaflet, Acute heart failure, Annuloplasty, Abdominal trauma, Paediatrics

## Abstract

**Background:**

Mitral valve injuries typically result from high-energy chest trauma, with occurrences following blunt abdominal trauma being exceptionally rare. To date, no cases of mitral valve injury caused by isolated blunt abdominal trauma in paediatric patients have been reported.

**Case summary:**

A 4-year-old boy presented with laboured breathing, tachypnoea, and tachycardia after sustaining localized blunt force abdominal trauma caused by an unintentional knee impact from an adult. Despite the absence of external signs of trauma and unremarkable findings on initial imaging, worsening symptoms and elevated cardiac biomarkers prompted echocardiography, which revealed severe mitral regurgitation due to a torn papillary muscle. Prompt surgical intervention, including chordal reattachment and mitral valve reconstruction with posterior ‘split’ annuloplasty, led to full recovery, with minimal residual regurgitation at 1-year follow-up.

**Conclusion:**

Isolated blunt abdominal trauma can result in severe cardiac injuries, even in the absence of clinical or radiographic signs of trauma. Early echocardiography and prompt surgical management were crucial for a favourable outcome.

Learning points

- The absence of overt clinical or radiographic signs after significant blunt abdominal trauma cannot rule out cardiac injuries with certainty.
- Although exceedingly rare, mitral valve injuries can occur following blunt abdominal trauma and should be considered in cases of persistent tachycardia and oxygen needs, especially when a systolic murmur is present.

## Introduction

Mitral valve injuries due to blunt trauma are rare, especially in paediatric patients. They typically result from high-energy chest trauma.^1,2^ To date, there are no documented cases of heart valve injuries following isolated blunt abdominal trauma in children. The severity and clinical manifestations of such injuries can vary, ranging from initially asymptomatic to rapidly progressing and life-threatening.^3–5^ This case report presents an extremely rare instance of severe mitral valve regurgitation following isolated abdominal trauma, complicated by a concomitant respiratory tract infection that obscured the diagnosis, ultimately resulting in heart failure requiring surgical intervention.

## Summary figure

**Table:** 

Admission to ED	Abdominal trauma. Preceding respiratory tract infection for several days. Presented with respiratory distress, tachypnoea, obstructive breathing pattern, tachycardia, and an unspecific systolic murmur (2/6)
Night of the third day	Persistent laboured breathing. Murmur louder and harsher. Chest X-ray reveals pneumonia. Unremarkable ECG. Elevated cardiac biomarkers. Echocardiography: severe mitral valve regurgitation with a flail posterior leaflet Transfer to intensive care unit Over next 2 days, deteriorating heart failure, inotropic support and mechanical ventilation, necessitating surgical intervention
Day 17	Discharge in stable condition

## Case presentation

A 4-year-old boy presented to the emergency department (ED) after being accidentally struck in the abdomen by his grandfather’s knee. The incident occurred when the grandfather stumbled and fell, applying his full body weight onto the child's abdomen, while the boy was lying supine on the floor. Prior to the abdominal trauma, the child had experienced a 7-day history of fever, cough, and laboured breathing. On arrival, he was tachypnoeic (44 breaths/min) with oxygen saturation of 85%, obstructive breathing, tachycardia (160 beats/min), normal blood pressure (93/61 mmHg), and an unspecific systolic murmur (2/6). Physical examination revealed no external signs of traumatic injuries, specifically no tenderness or haematoma over the abdominal or thoracic regions. On suspicion of obstructive bronchitis, treatment with oxygen, bronchodilators, and betamethasone was initiated.

**Figure 1:**
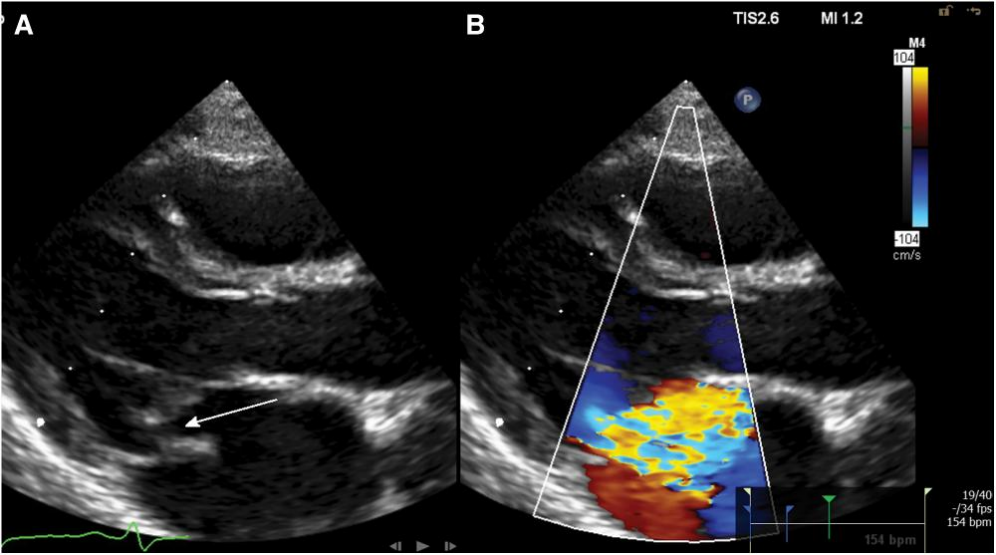
Transthoracic echocardiography, parasternal long-axis view showing *(A)* the mobile part of the torn papillary muscle in the left atrium in systole with resulting flail gap (arrow) and *(B)* severe mitral regurgitation on colour flow Doppler.

Over the next 2 days, his condition gradually worsened, with persistent tachycardia and increasing oxygen needs, reaching 3 L/min. An abdominal ultrasound and chest X-ray showed no signs of trauma-related sequelae or cardiopulmonary congestion but revealed a left upper lobe infiltrate. Elevated leucocyte counts (15.16 g/L) and procalcitonin (0.52 µg/L) suggested possible bacterial infection, and despite low C-reactive protein levels (CRP, 0.8 mg/L), oral amoxicillin was started after obtaining blood cultures. The systolic murmur became progressively louder and harsher. Suspecting myocarditis, further diagnostics were undertaken, including an electrocardiogram (ECG), which showed no indicative changes other than tachycardia. Troponin I (634 ng/L,<45 ng/L) and N-terminal prohormone of brain natriuretic peptide (10 102 ng/L, <85 ng/L) levels were significantly elevated. Transthoracic echocardiography (TTE) revealed severe mitral valve regurgitation with a flail posterior leaflet from a torn papillary muscle (Carpentier type II; *Figure 1* and Supplementary material online, *Videos S1*–*S3*).

**Figure 2:**
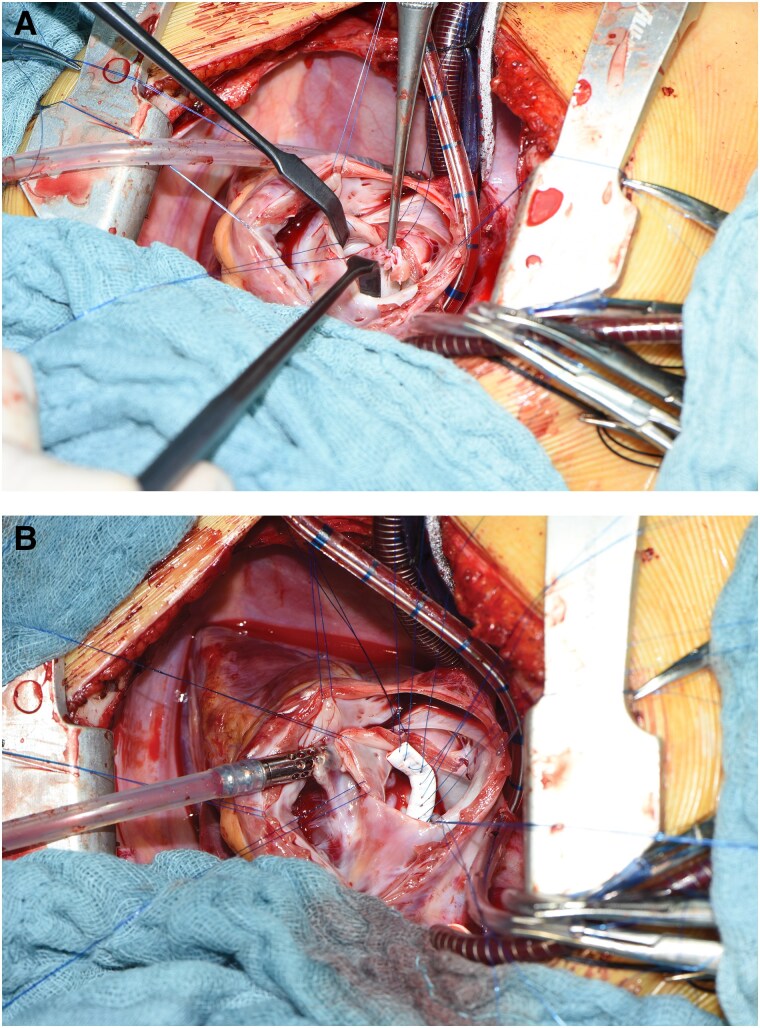
Intraoperative photographs: *(A)* showing the stump of the avulsed papillary muscle with its chorda from the postero-medial papillary muscle, and *(B)* showing posterior annuloplasty using a 3 mm Gore-Tex graft and multiple single 5.0 polypropylene stitches. The graft was split into two halves afterwards to allow for annular growth.

**Figure 3:**
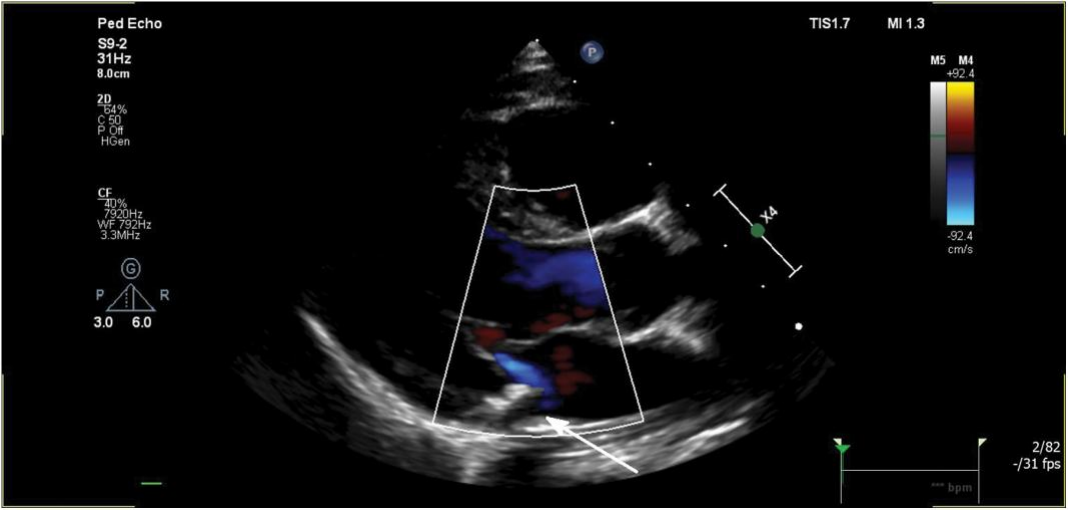
Transthoracic echocardiography, parasternal long-axis view, 15 months after mitral valve reconstruction. Minimal mitral regurgitation, the Gore-Tex ring can be appreciated as an echogenic structure (arrow).

The patient was transferred to the intensive care unit with heart failure, subsequently necessitating mechanical ventilation and inotropic support. A nasopharyngeal swab revealed an Influenza A infection, while blood cultures remained negative, leading to the discontinuation of antibiotic therapy. To rule out endocarditis, a transoesophageal echocardiography (TOE) was performed, with no evidence of vegetative lesions observed. Preoperative ECG was unremarkable and surgical repair of the mitral valve was performed, including refixation of the ruptured chordal tendineae, and a posterior ‘split’ annuloplasty using a 3 mm Gore-Tex tube prothesis (*Figure 2* and Supplementary material online, *Preoperative ECG S4*). Myocardial biopsy showed no signs of myocarditis. The patient was discharged on the 15th postoperative day. One-year follow-up echocardiography revealed minimal mitral regurgitation with no significant stenosis (*Figure 3*).

## Discussion

This case highlights that isolated blunt abdominal trauma can lead to severe cardiac injuries in children. Mitral valve injuries are often insidious in nature, presenting with subtle or delayed symptoms that require careful clinical evaluation and a high degree of suspicion.^3–5^

In general, post-traumatic valvular injuries, particularly isolated mitral valve injuries, are rare. These injuries are most commonly associated with blunt chest trauma, which predominantly affects the aortic valve, followed by the mitral valve, due to the higher pressures within the left heart.^6,7^ To date, no case of mitral valve injury secondary to isolated abdominal trauma in children has been reported.

The primary mechanism leading to mitral valve regurgitation after blunt chest trauma involves a sudden increase in intracardiac pressure during early systole, specifically during the isovolumetric contraction phase between the closure of the mitral valve and the opening of the aortic valve, leading to lesions in the papillary muscles, chordae tendineae, and mitral valve leaflets.^3,8^ Indirect trauma, such as injury to the abdomen or lower extremities, can cause a sudden rise in intra-abdominal pressure, displacing the viscera upward and generating a retrograde aortic pressure wave, a mechanism known as the ‘Hydraulic Ram’ or ‘Water Hammer’ effect. This phenomenon can lead to cardiac injuries, particularly affecting the valves on the left side of the heart.^9^ Additionally, cases involving pericardial injuries have also been described.^10^ It is possible that the trauma sustained by the child involved both abdominal and thoracic components. The absence of chest bruising or other external clinical and radiological signs indicative of thoracic trauma makes this less likely. While a thoracic component cannot be entirely ruled out, it remains speculative based on the available evidence.

An alternative explanation for primary mitral regurgitation could involve the degeneration of the papillary muscles or chordae tendineae secondary to myocardial ischaemia or infection.^11^ However, myocardial infarction is exceedingly rare in a healthy paediatric patient, and no supporting evidence was found on electrocardiography, echocardiography, or intraoperative assessment. Furthermore, no suspicious auscultatory findings had been documented during previous routine check-ups, and the patient's medical history revealed no indications of prior underlying cardiac pathology. While a preceding respiratory infection could theoretically spread to affect the heart, myocardial biopsy showed no signs of infection, and a TOE showed no signs of endocarditis. There were no clinical signs of connective tissue weakness predisposing to rupture of the papillary muscles or chordae, and new generation sequencing did not reveal any explaining abnormalities in >100 investigated connective tissue genes, further supporting a purely post-traumatic origin of the mitral valve regurgitation.

The child’s acute clinical decline following admission is likely due to an initial partial tear of the papillary muscle, which progressed to complete rupture during hospitalization. This sequence would explain the intensifying systolic murmur, rapid clinical deterioration, and markedly elevated cardiac biomarkers. An infectious aetiology is highly unlikely based on low CRP levels and negative blood cultures.

A detailed medical history and clinical examination should raise suspicion of cardiac injury, prompting further diagnostic evaluation with ECG, chest X-ray, blood tests, and echocardiography—the gold standard for diagnosing mitral valve injuries. In this case, TTE was crucial in identifying the underlying cause of symptoms.

For mitral valve injuries resulting in Carpentier type II mitral regurgitation, on-pump surgery with cardioplegia-induced cardiac arrest is usually performed.^12^ The posterior flail leaflet was reconstructed by reinserting the affected papillary muscle. To reinforce the slightly dilated posterior mitral valve annulus, which caused moderate mitral regurgitation, a posterior annuloplasty was performed. The Gore-Tex graft material used was split in two halves to accommodate for future growth.

## Conclusion

Although exceedingly rare, isolated blunt abdominal trauma can cause severe cardiac valve injuries. Concomitant conditions, such as the respiratory tract infection in this case, may delay critical diagnostic steps. The patient’s deterioration prompted expanded diagnostics, revealing severe mitral regurgitation caused by a torn papillary muscle, leading to timely and successful surgical intervention. This case highlights the importance of maintaining a high index of suspicion for cardiac injuries, even in the absence of overt trauma signs.

## Supplementary Material

ytaf474_Supplementary_Data

## Data Availability

The data underlying this article are available in the article and in its online Supplementary material.

## Lead author biography

Samuel Waser graduated from the University of Zurich, Switzerland, and is currently completing a residency in the Paediatric Department at the Children's Hospital Aarau, Switzerland. Poster presentations have been given at the annual conferences of the Swiss Society of Paediatrics and the German Society of Forensic Medicine. He has a clinical interest in paediatric cardiology and aims to pursue subspecialization in this field.
