# Supplementary figures and images for: Severe mitral valve regurgitation in a preschool boy following blunt abdominal trauma: a case report
Source: Eur Heart J Case Rep. 2025 Sep 19;9(10):ytaf474. doi: 10.1093/ehjcr/ytaf474 (PMC12559570; doi:10.1093/ehjcr/ytaf474)

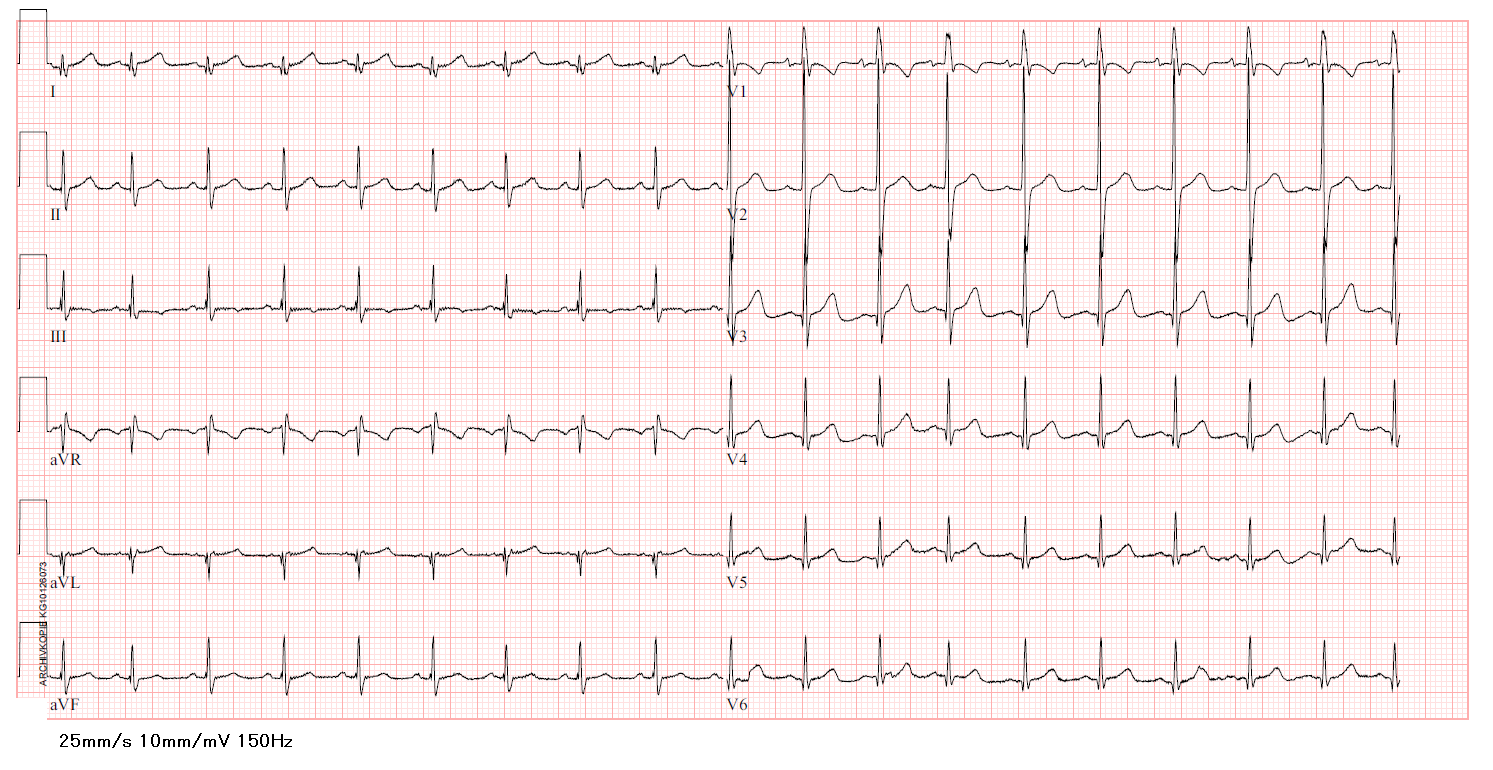

Supplement: ytaf474_Supplementary_Data [file ytaf474_supplementary_data.zip › Supplementary preoperative ECG.PNG]

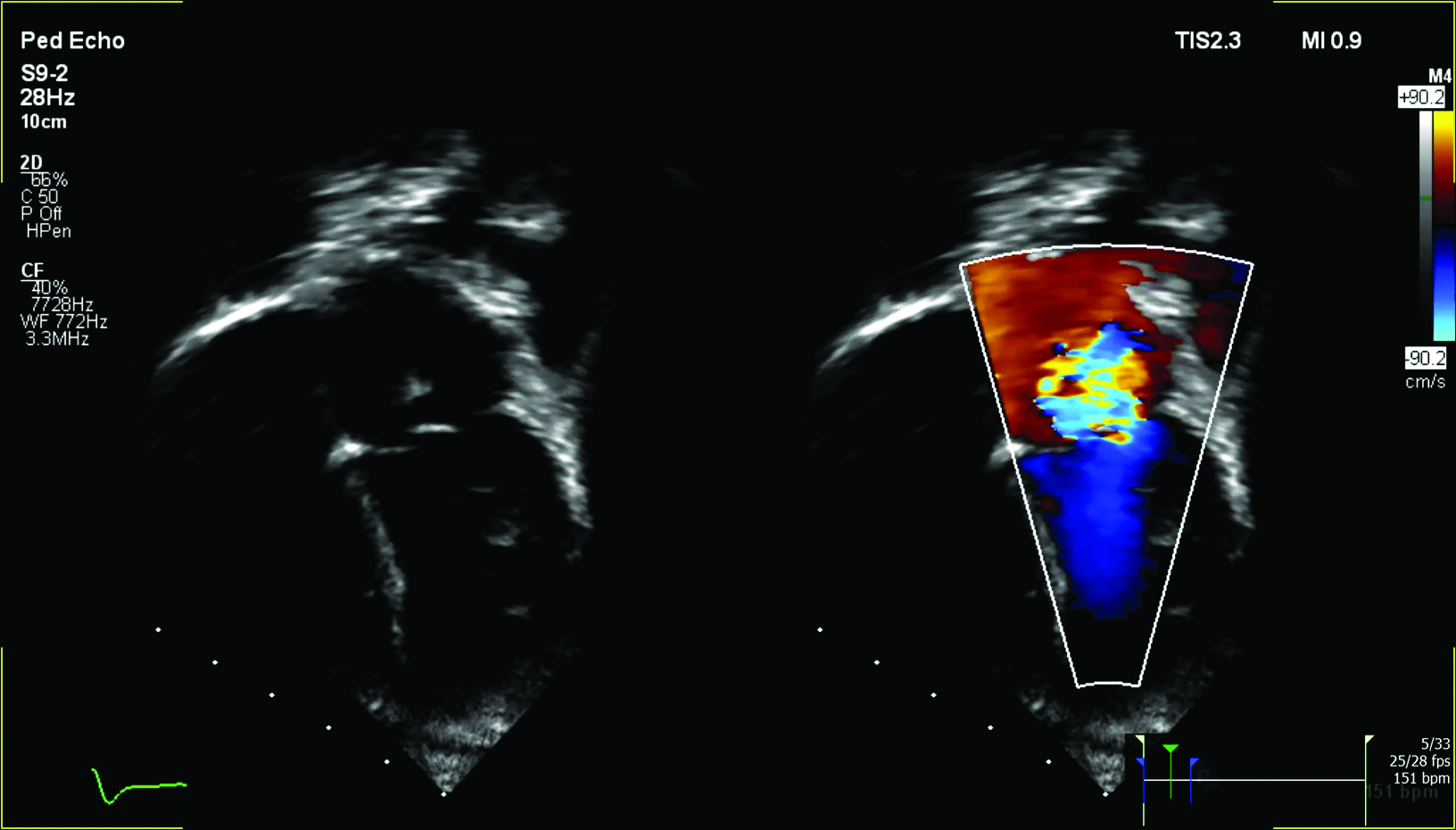

Supplement: ytaf474_Supplementary_Data [file ytaf474_supplementary_data.zip › Supplementary video S1 still image.tif]

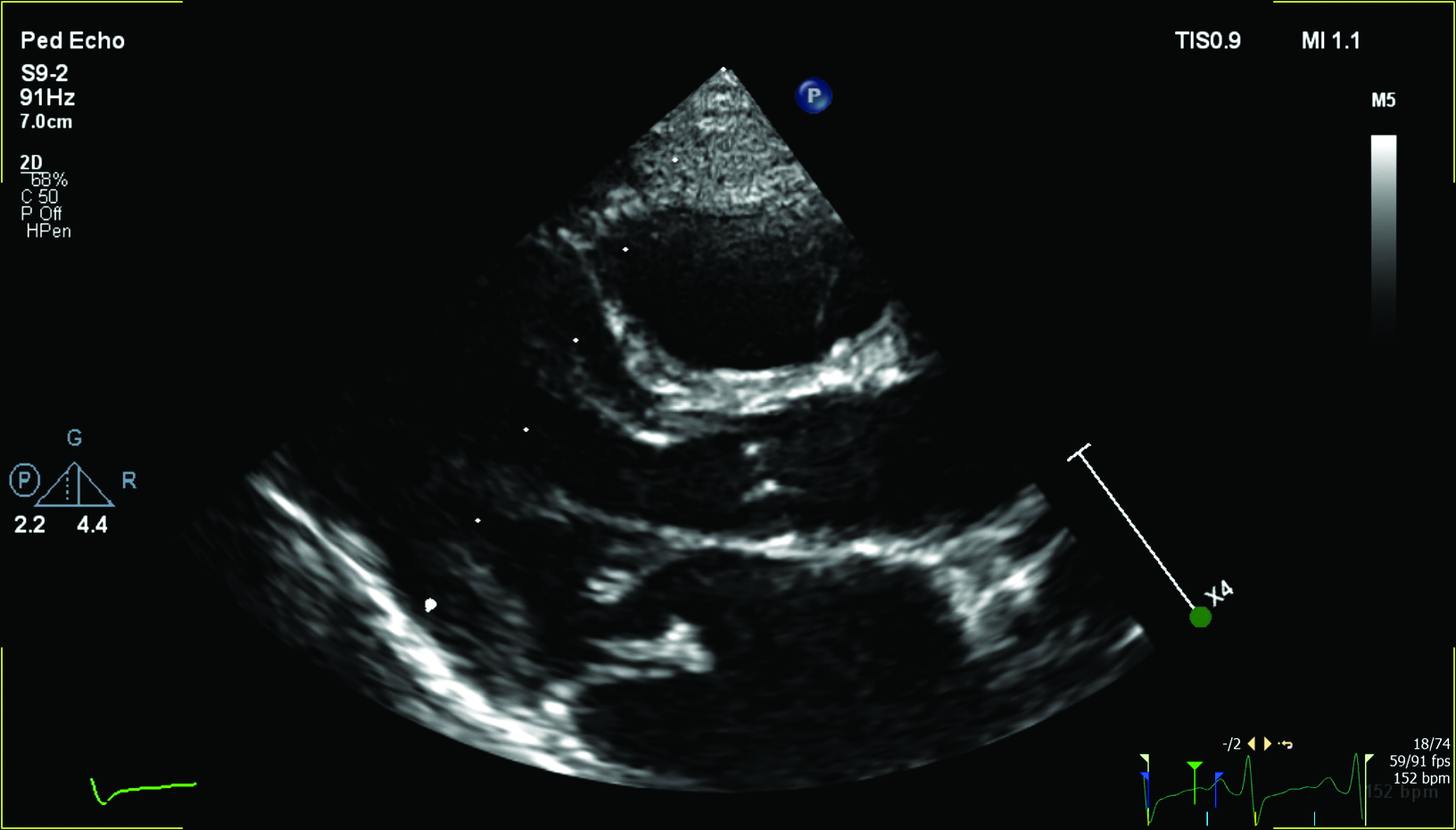

Supplement: ytaf474_Supplementary_Data [file ytaf474_supplementary_data.zip › Supplementary video S2 still image.tif]

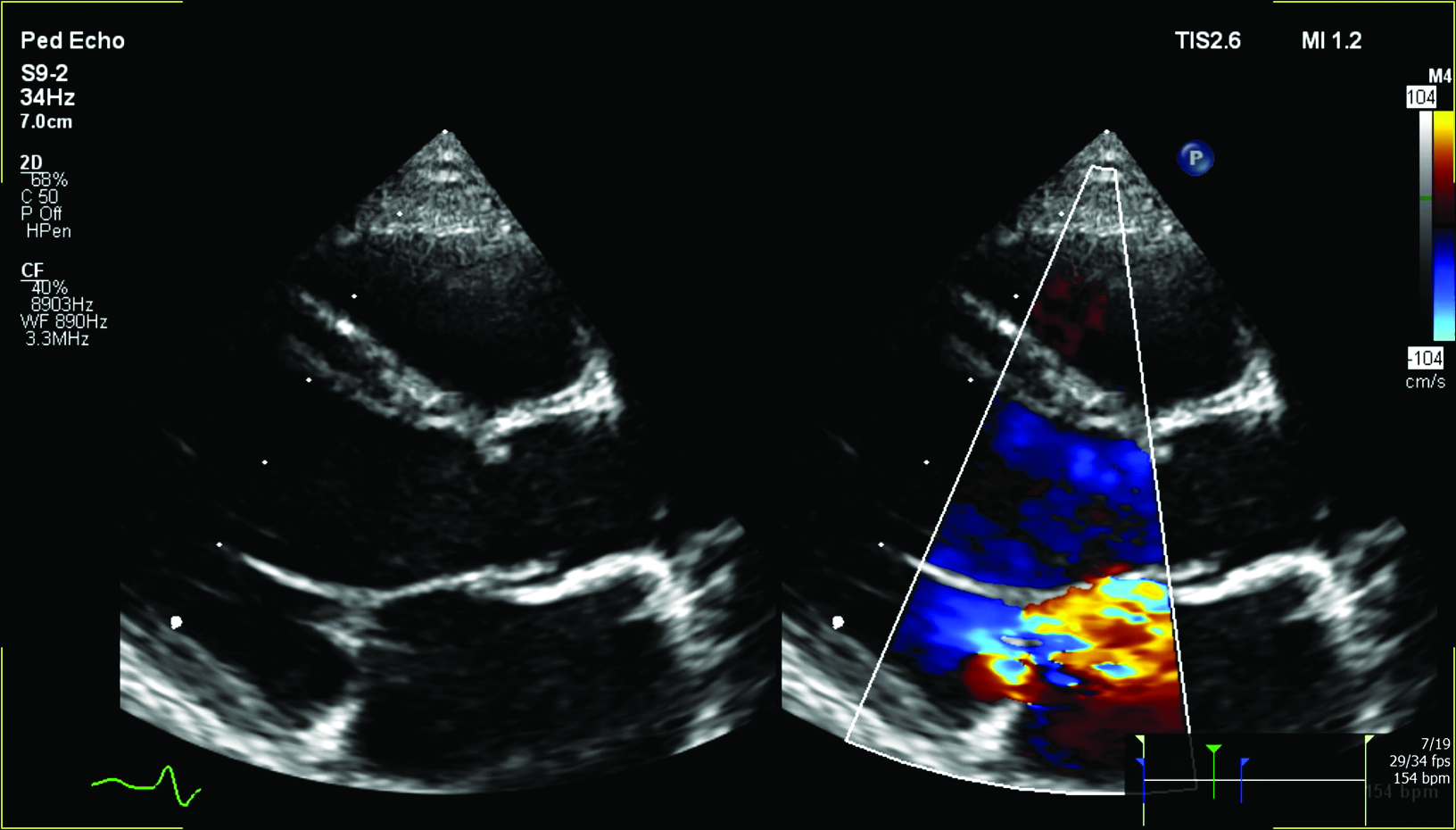

Supplement: ytaf474_Supplementary_Data [file ytaf474_supplementary_data.zip › Supplementary video S3 still image.tif]
